# The role of foam cells in spinal cord injury: challenges and opportunities for intervention

**DOI:** 10.3389/fimmu.2024.1368203

**Published:** 2024-03-13

**Authors:** Xiao-Xin Wang, Ze-Hui Li, Hua-Yong Du, Wu-Bo Liu, Chun-Jia Zhang, Xin Xu, Han Ke, Run Peng, De-Gang Yang, Jian-Jun Li, Feng Gao

**Affiliations:** ^1^ School of Rehabilitation, Capital Medical University, Beijing, China; ^2^ Department of Spinal and Neural Functional Reconstruction, China Rehabilitation Research Center, Beijing, China; ^3^ Cheeloo College of Medicine, Shandong University, Jinan, Shandong, China; ^4^ Institute of Rehabilitation Medicine, China Rehabilitation Research Center, Beijing, China

**Keywords:** spinal cord injury, macrophage, foam cell, scavenger receptor, cholesterol reverse transcription

## Abstract

Spinal cord injury (SCI) results in a large amount of tissue cell debris in the lesion site, which interacts with various cytokines, including inflammatory factors, and the intrinsic glial environment of the central nervous system (CNS) to form an inhibitory microenvironment that impedes nerve regeneration. The efficient clearance of tissue debris is crucial for the resolution of the inhibitory microenvironment after SCI. Macrophages are the main cells responsible for tissue debris removal after SCI. However, the high lipid content in tissue debris and the dysregulation of lipid metabolism within macrophages lead to their transformation into foamy macrophages during the phagocytic process. This phenotypic shift is associated with a further pro-inflammatory polarization that may aggravate neurological deterioration and hamper nerve repair. In this review, we summarize the phenotype and metabolism of macrophages under inflammatory conditions, as well as the mechanisms and consequences of foam cell formation after SCI. Moreover, we discuss two strategies for foam cell modulation and several potential therapeutic targets that may enhance the treatment of SCI.

## 1 Introduction

Spinal cord injury (SCI) induces primary damage to neurons and glia due to mechanical trauma, followed by secondary injury characterized by a cascade of inflammatory processes that cause further cell death of oligodendrocytes, astrocytes, and especially neurons (1). A key feature of the inflammatory response in SCI is the infiltration of monocyte-derived macrophages (MDMs) into the lesion site and the activation of resident microglia in the central nervous system (CNS). Both cell types act as major phagocytes that remove tissue debris after injury, which is crucial for creating a regenerative environment. It is well established that tissue debris from peripheral injuries is cleared by MDMs within weeks after injury. However, after SCI, the persistent demyelination produces myelin debris and other lipid-rich histiocyte debris that induce the transformation of macrophages into foamy macrophages, resembling those found in atherosclerotic plaques. These foam cells persist in the lesion site and mediate chronic inflammation, which hampers nerve repair (2, 3). T The molecular mechanisms of foam cell formation and function are poorly understood, but their phagocytic and migratory abilities have been reported to be impaired (2–6). Thus, foam cells after SCI represent a potential target for novel SCI research and intervention.

In this review, we provide a brief overview of the lipid metabolism of macrophages in the inflammatory milieu, and how it influences their function and phenotype. This will help to elucidate the mechanisms and consequences of macrophage phenotypic alterations after SCI. Furthermore, we summarise and discuss the factors that induce foam cell formation after SCI and the potential targets for modulating them, aiming to identify the strategies for foam cell regulation after SCI.

## 2 Macrophage metabolic regulation in inflammation

Different types of stimuli induce different macrophage phenotypes and metabolic profiles, and immune activation can be divided into four main components: inducers (signals that trigger an inflammatory response), sensors (proteins that detect the inducers), mediators (molecules that transmit signals to activate an effector response), and effectors (downstream metabolic programmes that facilitate the maturation of the desired effector phenotype) (7, 8). Based on this general classification, the macrophage response to stimulation can be described more clearly. The phenotypes that emerge from macrophage activation are commonly categorised as the M1 phenotype and the M2 phenotype. The M1 phenotype is mainly associated with acute bacterial infections, and the typical inducers are pathogen-associated molecular patterns (PAMPs), which are sensed by cell surface pattern recognition receptors (PRRs), leading to the production of the intermediate mediator, HIF-1α, which connects glycolytic metabolism to the inflammatory and microbicidal programmes of the macrophage (9). The M2 phenotype is driven by IL-4 and IL-13 produced by innate and adaptive immune cells (e.g., mast cells, basophils, and T cells) during processes such as helminthic and parasitic infections (10, 11). After IL-4 and IL-13 bind to their respective receptors (IL-4Rα vs. IL-13Rα), the subsequent activation of the M2 cells largely depends on STAT6, which is transcriptionally activated to upregulate fatty acid β-oxidation, OXPHOS and metabolic genes essential for mitochondrial biogenesis (12). Additionally, STAT6 induces the expression of transcriptional regulatory proteins and co-activator proteins, such as PPARγ, PPARδ and PGC-1β, which cooperate with STAT6 to sustain the switch of the cellular metabolic profile to oxidative metabolism (12–15). Different metabolic profiles of the M1 and M2 phenotypes stem from this, with the M1 macrophage using Warburg metabolism and the M2 macrophage using OXPHOS, which has been extensively discussed by others in the literature (16–19). The metabolism of M1 and M2 macrophages is bi-directionally linked to their functional phenotypes. Glycolysis in M1 macrophages enhances PPP flux, which provides intermediates for the biosynthesis of ribose, fatty acids and non-essential amino acids to sustain vital metabolic pathways. In contrast, the activation programme in M2 macrophages requires high levels of both duration and intensity, making the TCA cycle more prominent than glycolysis. Fatty acid oxidation is especially important in these cells to support the TCA cycle. One source of fatty acids is triglycerides, which are internalised by M2 macrophages via CD36 and then hydrolysed by lysosomal acid lipase (20). Besides uptake from external sources, macrophages also have the ability for lipid synthesis (21, 22). Microbial stimulation can augment macrophage *de novo* lipogenesis (DNL). Microbial stimulation also increases glucose utilisation, redirects intracellular glucose metabolism, and supplies a reaction substrate for intracellular lipid regeneration (DNL) in macrophages (23, 24). On the other hand, it can also activate the signalling pathway triggered by the transcription factor NF-κB, which directly binds to the response element in the promoter of SREBP1a (an isoform expressed in macrophages), thereby enhancing the expression of SREBP1a, which in turn promotes the oxidation of fatty acids in the DNL, providing nutrients for the TCA cycle (25). The inhibition of lipolysis reduces M2 macrophage-mediated resistance to parasite infection (26). These metabolic changes are not irreversible. Unlike metabolic shifts in cancer (genetically driven aerobic glycolytic transitions) or slow exercise-induced myofibre type conversions in skeletal muscle, many immune cells can swiftly switch between glycolysis and OXPHOS in response to external signals.

It is noteworthy that some vitro studies have proposed that the key determinant of the two phenotypes is a difference in the metabolic pathways used by the cells, i.e. the use of metabolic substrates controls macrophage polarization. However, this conclusion is disputed, with Chang HR et al. demonstrating that cellular lipid metabolism does not correlate with macrophage phenotype, and that the tissue environment is the main factor in determining how subpopulations of macrophages respond to changes in lipid synthesis and metabolic capacity (27). Furthermore, although the M1 and M2 phenotypes of macrophage activation facilitate the understanding of macrophage responses to stimuli, the *in vivo* phenotype of macrophages is more complex, especially in spinal cord injury (28).

## 3 Mechanisms and consequences of foam cell formation after spinal cord injury

**Figure 1:**
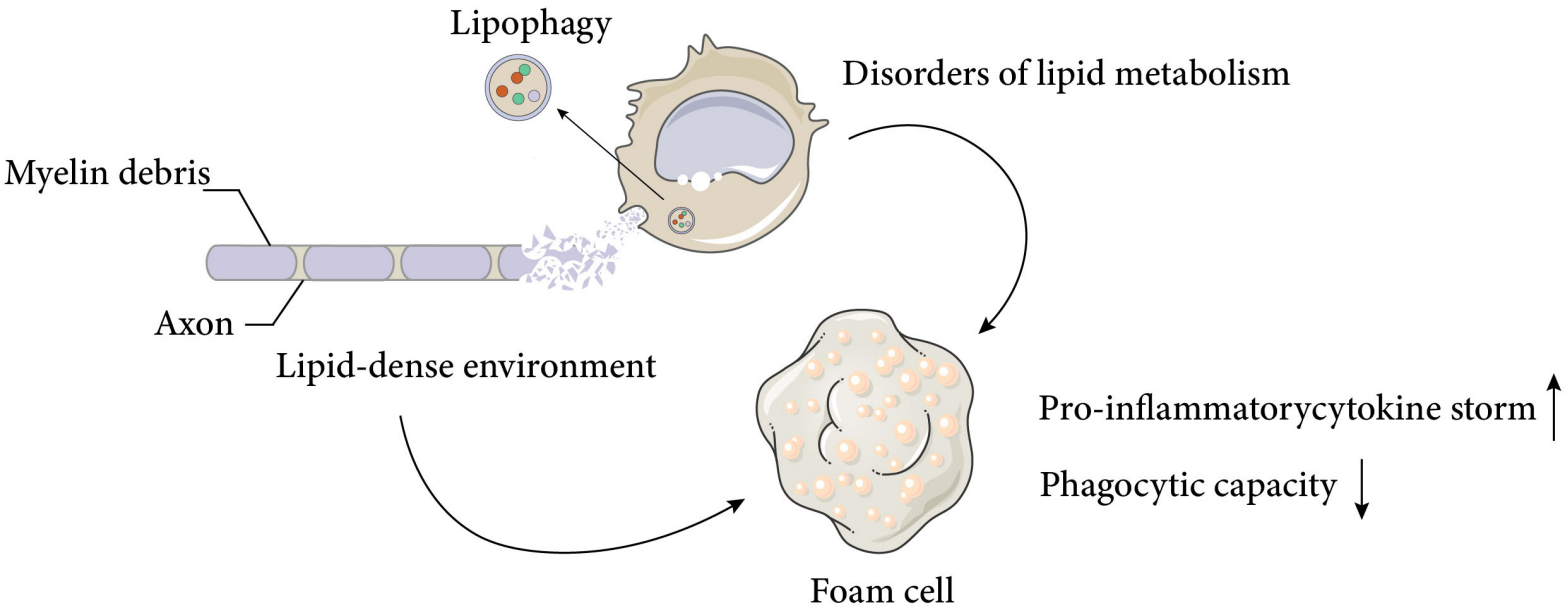
Causes and negative effects of foam cell formation after spinal cord injury. Spinal cord injury results in massive cell death and the accumulation of cellular and myelin debris at the lesion site, creating a lipid-rich environment. Macrophages can phagocytose these debris and promote a regenerative milieu that stimulates axonal myelination and repair. However, chronic exposure to the lipid-rich environment and impaired cellular lipid metabolism lead to the formation of foam cells, which produce pro-inflammatory cytokines and exhibit reduced phagocytic activity. These factors adversely affect neural recovery after spinal cord injury. The Figure was partly generated using Servier Medical Art, provided by Servier, licensed under a Creative Commons Attribution 3.0 unported license.

As discussed above, macrophages display various functional states that influence tissue repair, and understanding the basic macrophage metabolism in inflammation will help to elucidate the complex changes that occur in macrophages after SCI. It is well known that the surrounding microenvironment is a key determinant of macrophage function (29). The spinal cord microenvironment undergoes drastic changes shortly after SCI. The early microenvironmental changes in SCI are characterised by an inflammatory response that induces monocytes to migrate to the injury site and differentiate into macrophages, which further amplify the inflammation. Inflammation after SCI is complicated and involves multiple cell types and a variety of inflammatory cytokines, such as tumour necrosis factor α (TNFα), interleukin-1β (IL-1β) and interleukin-6 (IL-6). Despite some beneficial effects of inflammation after SCI, excessive infiltration of immune cells is the main cause of neurodegeneration. After the acute inflammation resolves, a large number of cells undergo apoptosis, generating large amounts of cellular debris, particularly myelin debris. The accumulation of myelin and cellular debris at the injury site, combined with the pre-existing CNS glial environment, forms an inhibitory environment in the centre of injury, which impairs nerve repair processes, such as oligodendrocyte precursor cells (OPCs) differentiation and synapse regeneration (30, 31). By removing myelin debris, lipid peroxidation of myelin-derived lipids in the injury centre is prevented and the inhibitory microenvironment is partially deregulated. Previous studies have demonstrated that the function of macrophages after SCI is significantly influenced by myelin debris, depending on the type and timing of stimulation, but this issue remains highly contentious (2, 32, 33).. Membrane lipids are classified into three major groups: cholesterol, phospholipids (e.g., plasminogen, lecithin, sphingomyelin), and glycolipids (e.g., galactose ceramide). Unlike most biological membranes (25%:65%:10%), myelin has a distinctive lipid composition, with a high proportion of cholesterol and glycolipids in a 40%:40%:20% ratio (cholesterol, phospholipids, and glycolipids) (34), and a total lipid dry weight of 70-75% (35, 36). This creates a unique lipid-rich environment. Dysfunctional lipid metabolism in macrophages, coupled with the lipid-rich environment, leads to the accumulation of intracellular lipids in macrophages, resulting in the formation of cell types resembling the specialised macrophages called foam cells in atherosclerotic plaques (**Figure 1**), which are also referred to as foam cells after spinal cord injury. Foam cell is a term widely used in the study of diseases such as atherosclerosis and tuberculosis. Foam cell formation is induced by various factors, such as uncontrolled uptake of modified low-density lipoprotein (LDL), increased cholesterol esterification, and impaired cholesterol release mechanisms (37). Macrophages internalize modified lipoproteins via surface scavenger receptors (SRs) like CD36 and SR-A. These SRs are macrophage PRRs that recognize and bind oxidized LDL, thereby promoting foam cell formation by internalizing these lipoproteins. Coated-pit endocytosis, phagocytosis and pinocytosis also mediate lipoprotein internalization. After internalization, cleared lipoproteins are transported to endosomes or lysosomes for degradation, where cholesteryl esters (CE) are hydrolyzed by lysosomal acid lipase (LAL) to unesterified free cholesterol (FC). Free cholesterol is transported to the endoplasmic reticulum where it is re-esterified by acyl-CoA: cholesterol acyltransferase 1 (ACAT1) and stored as cytoplasmic lipid droplets. These lipid droplets look like bubbles within macrophages, hence the name foam cells. The foam cell formation process above has been detailed in articles (38, 39). It is noteworthy that while most studies have focused on macrophages as the main or only source of foam cells, the role of smooth muscle cell-derived foam cells in atherosclerosis has gained more attention (40). This article focuses on macrophage-derived foam cells, but the reader should be aware that foam cells are defined by their morphology and not by their precursor cells.

These foam cells are typically observed in the injury area 1 week after SCI in mice and persist for at least 4 weeks (2).. Early studies suggested that the uptake of myelin residues biased macrophage polarisation towards a beneficial anti-inflammatory phenotype (41), but more recent studies have shown that foamy macrophages in SCI, which lose their ability to phagocytose apoptotic cells and tissue debris, activate NF-κ B signalling, which promotes inflammation and may contribute to further neurodegeneration (2, 3). The direction and modes of intervention of foam cells will be discussed later.

## 4 Possible strategies for reducing foam cell formation

**Figure 2:**
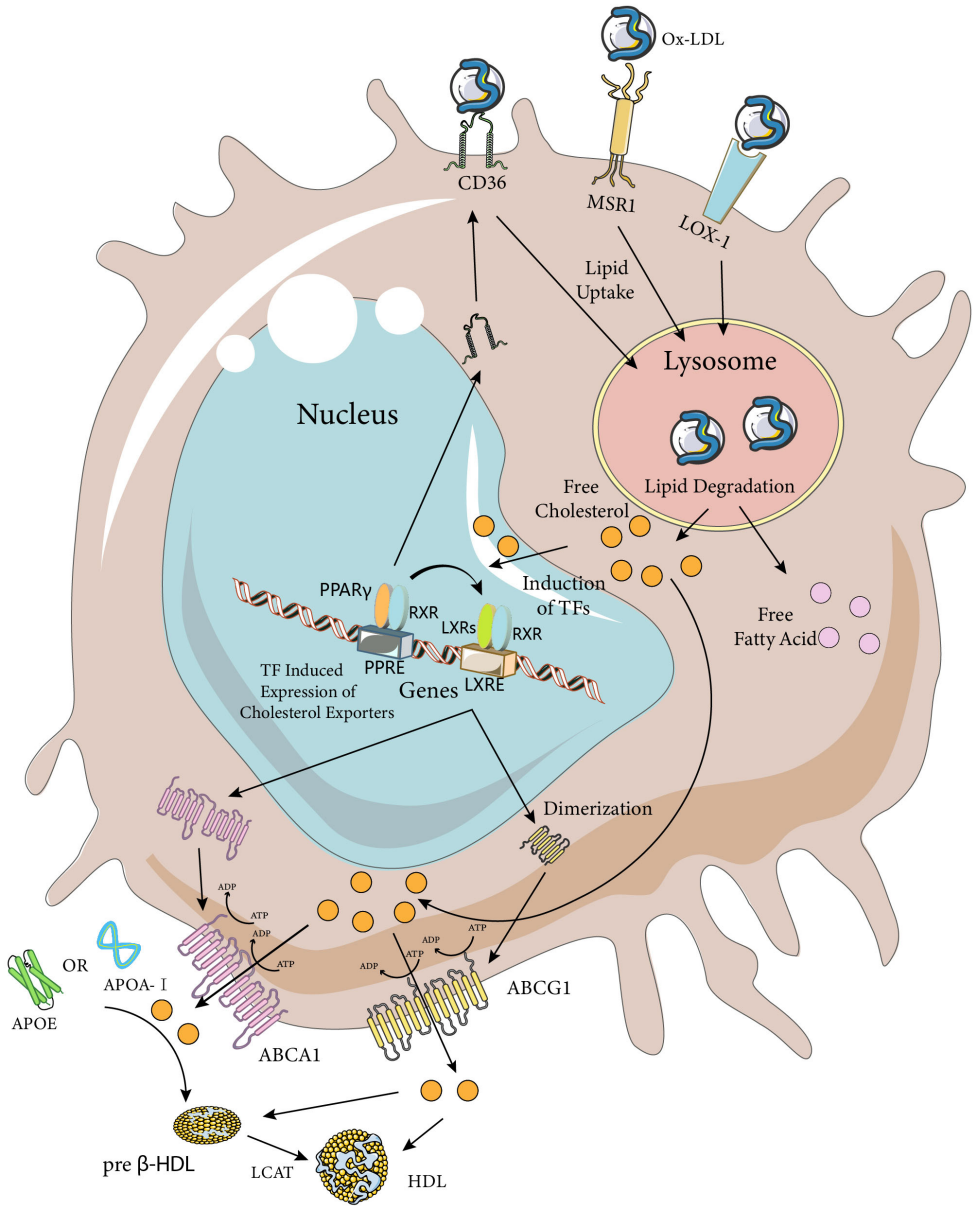
Macrophage Ox-LDL uptake and cholesterol efflux pathways. Macrophages internalize modified LDL (mainly Ox-LDL) via MSR1, CD36, and LOX-1 receptors. Ox-LDL is degraded in lysosomes to release lipids such as free cholesterol and fatty acids. Excess cellular cholesterol activates several transcription factors, including PPARγ, LXRs, and RXRs. Activated PPARγ and LXRs upregulate the expression of their target genes by heterodimerizing with RXRs and binding to the PPARE and LXRE response elements, respectively. The PPARγ transcriptome further enhances the expression of LXRs and CD36, while the LXR transcriptome induces the expression of the cholesterol efflux transporters ABCA1 and ABCG1. ABCA1 is a full-length membrane transporter, while ABCG1 functions as a dimer. Both proteins have two nucleotide-binding and two transmembrane domains and use ATP hydrolysis to transport substrates. ABCA1 mediates the efflux of cholesterol (chol) and phosphatidylcholine (PC) to ApoA-I or ApoE, forming preb-HDL, and ABCG1 mediates the efflux of cholesterol, sphingomyelin (SM), and phosphatidylcholine to preb-HDL or HDL (only the cholesterol transporter portion is highlighted in the figure). Cholesterol in preb-HDL and HDL is esterified by lecithin-cholesterol acyltransferase (LCAT) to form mature HDL. The Figure was partly generated using Servier Medical Art, provided by Servier, licensed under a Creative Commons Attribution 3.0 unported license (https://creativecommons.org/licenses/by/3.0/).

Pathway analysis of genes involved in macrophage lipolysis metabolism revealed that lipolysis metabolic processes were a major function of macrophages seven days after spinal cord injury (compared to three days after spinal cord injury), with hubs including TNF, CD36, LPL, PPARγ, and ABCA1, among which the LXR/RXR pathways (CPs) were considered to be one of the most enriched pathways (42). Except for TNF expression, which was decreased, all gene-enriched pathways were increased, and the above receptors and pathways involved in phagocytosis of tissue debris and lipid efflux might be a key direction in dealing with foam cells (**Figure 2**). To discuss the direction of foam cell intervention, we first need to clarify the functional differences between MDMs and microglia after spinal cord injury. Recent lineage tracing studies have indicated that almost all tissue-resident macrophages in adult mice originated from embryonic yolk sac progenitor cells and that their numbers were maintained by local proliferation throughout the organism’s life cycle (43–45). It is worth noting that there was confusion about the distinction between MDMs and microglia in previous studies due to the difficulty in distinguishing them from each other. The two types of cells showed similarities in morphology as well as gene and cell surface protein expression after activation, but the two types of cells differed in the timing of activation, location, and efficiency of processing of myelin debris after SCI. After SCI, as a result of primary mechanical injury and secondary death due to apoptosis, the number of microglia at and near the site of injury was significantly reduced (46). However, surviving microglia responded rapidly to ATP released by damaged cells. They also responded to danger and pathogen-associated molecular patterns (D/PAMPs) by releasing inflammatory molecules and were in turn affected by inflammatory molecules. Once activated, microglia could proliferate, migrate, and perform various effector functions including phagocytosis and proliferation of neuroinflammation by secreting additional chemokines and cytokines, and had better myelin debris processing efficiency compared to MDMs (46, 47). However, MDMs that subsequently infiltrated into the injury area inhibited microglia phagocytosis and gradually replaced microglia as the primary phagocytes after injury (48). Although the reason why microglia were less susceptible to foam cell formation remained to be explored, their potential as a target for intervention in the regulation of lipid metabolism after spinal cord injury had been well demonstrated. Since MDMs were the main phagocytes that formed foam cells, the potential interventions discussed subsequently would mainly target MDMs cells.

### 4.1 Decreased lipid phagocytosis by macrophages

As mentioned earlier, a major cause of foam cell formation after SCI is the high lipid content of the tissue debris and cells phagocytosed by macrophages. One study found that about half of the neutral lipids in foamy macrophages originated from non-myelin sources by culturing macrophages with spinal cord homogenates, suggesting that the conclusions of *in vitro* experiments on myelin-induced foamy cells might be controversial and heterogeneous (49). Therefore, modulating macrophage phagocytosis to reduce myelin and other lipids could be a way to reduce foam cell formation. Among the various subtypes of the scavenger receptor (SR) family, SR-A type I and II, CD-36 and LOX-1 have been found to be involved in foam cell formation through uptake of modified LDL (**Figure 2**) (50). *In vitro* studies have shown that SR-A and CD36 on macrophages account for up to 75% to 90% of oxidized low-density lipoprotein (Ox-LDL) or acetylated low-density lipoprotein (Ac-LDL) uptake, and both have been extensively studied in spinal cord injury (51). LOX-1 has been less studied in relation to spinal cord injury, but its role in foam cell formation has been systematically investigated *in vitro* and in diseases such as atherosclerosis. Therefore, a detailed discussion of the phagocytosis and regulation of foam cells mediated by these three scavenger receptor subtypes in spinal cord injury will follow. It is worth noting that myelin sheaths and associated tissue debris after spinal cord injury have an inhibitory role in neural repair, and the formation of foam cells after phagocytosis of tissue debris by macrophages can also have a negative role in neural repair, implying that simply decreasing or increasing macrophage phagocytosis may not be a perfect and reasonable intervention. Nevertheless, several studies manipulating macrophage phagocytosis have obtained positive results. This may be attributed to various factors, such as the phagocytic capacity of different cell types within the CNS. Macrophages, as “specialized” phagocytes, mediate the phagocytosis of most tissue debris after spinal cord injury. However, some “non-specialized” phagocytes (e.g., fibroblasts, astrocytes, neurons, and oligodendrocytes) also contribute to this process. In addition, macrophage phagocytosis-associated receptors may have multiple functions that require further exploration.

#### 4.1.1 CD36

CD36 is a type 2 cell surface scavenger receptor widely expressed on many immune and non-immune cells. It acts as both a signalling receptor in response to DAMPs and PAMPs and a long-chain free fatty acid transporter. Recent studies have indicated that CD36 can integrate cell signalling pathways and metabolic pathways through its dual functions, thereby influencing immune cell differentiation and activation, and ultimately cell fate. CD36 recognises and binds with high affinity to Ox-LDL and to specific oxidised phospholipids fractions on the surface of some apoptotic cells (52–54). Binding and uptake of ox-LDL by human macrophages lacking CD36 is reduced by 40% (42). Knockout of CD36 in spinal cord injured mice resulted in reduced lipid droplet content in macrophages, reduced foamy macrophage formation, reduced area of injury, and better functional recovery compared to wild-type mice (42, 55). CD36 deletion can also lead to a reduction in vascular ERSR, which improves functional recovery after spinal cord injury (55). A recent study showed that CD36 is expressed in fibroblasts, a major cellular component of glial scar formation in the spinal cord. Jun is a key factor in the formation of pathologic cutaneous scarring, and CD36 is a downstream effector (56). Increased expression of Jun in fibroblasts after SCI and induced expression of CD36 activated fibroblasts to form in the mouse scarring, implying that CD36 is one of the regulators of the glial scarring response after SCI (57). CD36 knockout improves the area of injury at 1 and 3 days post-injury in terms of vascular supply, it is noteworthy that the angiogenic response after spinal cord injury in wild-type mice does not begin until 3 dpi (58), so this early vascular improvement may be due to increased vascular retention or it may be related to an increase in vascular perfusion within the injured area as a result of the deregulation of the negative regulatory effect of CD36 on endothelial cell nitric oxide synthase and cyclic guanosine monophosphate (cGMP) production (59, 60). Notably, the role of CD36 for endothelial cells and angiogenesis and vascular repair remains unclear, with studies showing that blockade of the CD36 signalling pathway increases proliferation of endothelial cells from the CNS (61), with an improved angiogenic response consistent with the known antiangiogenic function of CD36, and studies showing that endothelial cell CD36 deficiency prevents normal angiogenesis and vascular repair (62). CD36 antagonists have been extensively explored. For example, salvianolic acid B, curcumin, and sulfosuccinimidyl oleate have been found to act as CD36 antagonists (63). In addition to these well-defined molecules, herbal extracts such as shibaosaponin A, epimedium glycosides, andrographolide, resveratrol, and quercetin have also been shown to modulate CD36 (57, 64, 65).

In summary, blocking the CD36 signalling pathway may reduce spinal cord injury lesion size, attenuate inflammation, decrease vascular ERSR, enhance acute heterotopic vascular proliferation and microvascular perfusion, improve scarring, and diminish foamy macrophage formation. These effects suggest that CD36 may be a promising target for SCI intervention.

#### 4.1.2 CD204-MSR1 (scavenger receptor A - SR-A)

Macrophage scavenger receptor 1 (MSR1) belongs to the scavenger receptor (SR) family and has three isoforms (1, 2, and 3) that are generated by alternative splicing of the MSR1 gene. These isoforms have distinct functions, but they share a common structure as macrophage-specific trimeric integral membrane glycoproteins. To avoid confounding effects, the study of MSR1 as a whole will be addressed in subsequent studies, but researchers should consider the functional differences among the isoforms when investigating MSR1. MSR1 is a receptor that is mainly expressed on immune cells and vascular endothelial cells, and it recognises and phagocytoses modified LDL. When LDL undergoes modifications, such as oxidation or glycosylation, within the vascular wall, it is recognised by MSR1 and taken up by macrophages, resulting in foam cell formation due to the accumulation of intracellular cholesterol. This is considered a key step in the pathogenesis of atherosclerosis, as it triggers the secretion of chemokines and pro-inflammatory cytokines (66, 67). Similar to other SRs, MSR1 binds, internalises and degrades various ligands, including heat shock proteins, surface molecules of bacteria and viruses (68, 69). These ligands are foreign substances that are recognised by the innate immune system, thus MSR1 acts as an innate pattern recognition receptor that mediates cellular processes such as host defence, phagocytosis and apoptosis. Besides its role in the immune response, MSR1 also participates in cellular functions such as lipid metabolism and cell adhesion, thereby influencing the functional regulation of several organs and diseases. For instance, MSR1 has been implicated in Alzheimer’s disease, tumours, myocardial infarction, coronary artery disease, etc., with either beneficial or detrimental effects. MSR1 is a receptor of broad biological significance, as it plays an important role in the development and progression of various diseases (70–74). Reichert et al. reported that MSR1 facilitates the phagocytosis of peripheral nerve myelin fragments *in vitro* (75). In spinal cord injury mice, the knockout of the MSR1 gene did not affect the macrophage infiltration in the injury area compared to wild-type mice, but MSR1 WT mice had a large number of myelin debris patches in macrophages. This suggests that the formation of foamy macrophages is restricted in mice lacking MSR1, and that MSR1 promotes foamy macrophage formation and exacerbates the detrimental effects of spinal cord injury. Since the phagocytosis of myelin debris by macrophages is closely related to lipid metabolism, further experiments are required to verify whether MSR1 influences the metabolism and efflux of myelin lipids (76).

#### 4.1.3 LOX-1

Lectin-like oxidised low-density lipoprotein receptor-1 (LOX-1) is also known as the Ox-LDL receptor. Ox-LDL interacts with the transmembrane glycoprotein LOX-1 and affects various cell types, such as endothelial cells, platelets, macrophages, fibroblasts and smooth muscle cells (77). Under normal conditions, LOX-1 expressed in macrophages accounts for 5-10% of Ox-LDL uptake. However, in pro-inflammatory states, LOX-1 expression is increased and contributes to approximately 40% of Ox-LDL turnover in macrophages (78, 79). Abnormal conditions, such as activation of pro-inflammatory pathways, hyperglycemia and hypertension, enhance LOX-1 expression and Ox-LDL uptake, leading to lipid accumulation and foam cell formation (80, 81). Several studies have shown that inhibition or down-regulation of macrophage LOX-1 expression reduces foam cell formation and lipid accumulation (82–84), suggesting its potential role in regulating foam cell formation and inflammatory factor expression in SCI. LOX-1 receptor expression is elevated at 14 days after spinal cord injury (85), but the expression pattern of LOX-1 at different time points after spinal cord injury is not well defined, and there is a lack of studies on the therapeutic effects of LOX-1 receptor intervention on SCI. This may be because the two receptors, CD36 and CD204, have received more attention, while the role of LOX-1 receptor has been overlooked. In conclusion, the observation and study of the expression and regulation of LOX-1 receptors may be important for understanding the formation of foam cells in the injury centre after spinal cord injury.

### 4.2 Enhancement of reverse cholesterol transport mechanisms in macrophages

Foam cells are formed when macrophages ingest excessive amounts of lipids after spinal cord injury. The prolonged persistence of foam cells after spinal cord injury indicates that the lipids accumulated in foam cells are not properly cleared (2). The persistence of foam cells impairs the subsequent phagocytic activity of macrophages and mediates chronic inflammation and poor neurological recovery. Therefore, besides regulating macrophage phagocytosis, enhancing cholesterol efflux from macrophages in the spinal cord injury site may be a strategy to address foam cell formation and accumulation. Generally, there are four cholesterol efflux pathways from cells: simple diffusion, scavenger receptor B1 (SR-BI, which belongs to the same family as CD36 but has different functions) mediated facilitated diffusion, and ATP-binding cassette transporters A1 (ABCA1) and G1 (ABCG1) in cooperation with extracellular lipid-poor apolipoproteins or more mature high-density lipoprotein (HDL) mediate cholesterol efflux (86). The role of SR-B1 in macrophage cholesterol efflux remains unresolved and is beyond the scope of this review. Here, we focus on ABCA1 and ABCG1, as well as apolipoprotein A-I (ApoA-I) and apolipoprotein E (ApoE), which are key mediators of cellular cholesterol efflux (**Figure 2**). We also discuss their relevance to spinal cord injury, where some studies have investigated their roles. It is noteworthy that two key issues should be considered when investigating cellular cholesterol efflux: the first is the existence of species variability in the cholesterol efflux pathway, which implies that the same receptor mediating cholesterol efflux may have different roles in different species; and the second is that the blood-brain barrier separates the CNS from the periphery, which allows for a different mechanism of cholesterol metabolism and cycling in the CNS compared to that of the peripheral tissues, such as the different structure and apolipoprotein content of HDL particles. The differences between the two should be taken into account when choosing and performing interventions.

#### 4.2.1 ABCA1 and ABCG

ABCA1 is an intact membrane transporter protein, ABCG1 is a half-transporter and needs to function as a dimer biologically. At the cellular level, macrophage-like cell lines from mouse or human origin consistently express ABCA1 and ABCG1 proteins, which are both important for maintaining cellular cholesterol homeostasis (87). The basal levels of ABCA1 and ABCG1 proteins are low in macrophages, but they are induced by cholesterol loading and modulated by HDL-mediated cholesterol efflux (88–90). Reverse cholesterol transport (RCT) is one of the major pathways for the removal of excess cholesterol from tissues (91). In this process, ABCA1 mediates the initial transport of cellular cholesterol to ApoA-I to form nascent HDL particles, and ABCG1 facilitates the subsequent sustained transport of cholesterol to HDL for further maturation (92). When mouse peritoneal macrophages are fully loaded with cholesterol and treated with diluted human serum, ABCA1 mediates approximately half of the cholesterol output and ABCG1 mediates 20% of the cholesterol output (93). Considering the critical role of ABCA1 and ABCG1 in mediating intracellular cholesterol export, combined up-regulation of the expression of these two transporter proteins may be more effective in inhibiting foam cell formation. LXR, a member of the nuclear receptor superfamily, plays a key role in the stimulation of ABCA1 transcription. LXR binds to RXR to form a specialized heterodimer. The LXR/RXR complex then binds to the promoter region of the ABCA1 gene, inducing its expression and playing a similar role to ABCA1 during ABCG1 transcription, a good way to regulate the expression of both proteins. It is noteworthy that the expression of ABCA1 in macrophages at the lesion site was significantly reduced after 7 days of spinal cord injury, suggesting a decrease in the capacity of macrophage RCT (2), and that this down-regulation is associated with the formation of foam cells, whereas the expression of the ABCG1 protein after spinal cord injury is still unknown. Similarly, several studies have observed and regulated the expression of ABCA1 protein after spinal cord injury: biosignature analysis showed that ABCA1 is a key gene closely associated with immune cell infiltration and may contribute to the pathogenesis of ischemic or hypoxic SCI by regulating vascular injury, inflammation, and immune infiltration (94). Atorvastatin indirectly upregulates ABCA1 through activation of PPAR-γ and promotes functional recovery (95). Allicin activates peroxisome proliferator-activated factor receptor (PPAR-γ), an inducer of ABCA1, stimulating macrophage lipid efflux and reducing foam cell formation (96). Whereas little research has been done on ABCG1 protein after spinal cord injury compared to ABCA1 protein, which may be related to the species-specific variability of its cholesterol efflux action, the important role of ABCG1 protein in RCT implies that it is likely to be one of the key targets for foam cell intervention after spinal cord injury.

#### 4.2.2 Apolipoprotein A-I

As discussed above, ApoA-I is a protein component of HDL that mediates reverse cholesterol transport. Although it is present in cerebrospinal fluid (CSF), its mRNA is not detected in the CNS, suggesting that ApoA-I is not synthesised in the brain and that it can enter the CNS with HDL via SR-BI (belonging to the CD36 family) mediated uptake (97). In contrast to ApoJ, ApoD, ApoA-II and ApoA-IV, which are present in the CNS in low abundance, ApoA-I (0.3.7 ± 0.0.8 mg/dl) and ApoE (0.3 ± 0.2.1 mg/dl) are the most abundant apolipoproteins in the CSF (98–100). ApoA-I has been shown to have a role in CNS inflammation and myelin regeneration (101–104). After spinal cord injury, ApoA-I expression is upregulated in injured spinal cord tissues (105). PPI network analysis showed it to be one of the 10 most altered core proteins 1 week after SCI (106), and changes in ApoA-I in the cerebrospinal fluid correlate with the severity of SCI injury, implying that ApoA-I may be a potential target for intervention after spinal cord injury (107). Monocytes and macrophages have the ability to synthesise and secrete endogenous ApoA-I (108). Two lipid-poor ApoA-I molecules bind ABCA1 dimers, allowing the transport of lipids in macrophages as high-density lipoproteins (HDL) (109). Recombinant exogenous human ApoA-I in mouse macrophages increased cholesterol efflux from macrophages and reduced the development of atherosclerosis, suggesting that macrophage-specific ApoA-I expression plays an important role in the prevention of atherosclerotic disease (110–112). Similarly, ApoA-I may be important for processing intracellular lipids in macrophages after spinal cord injury. Currently, there is still no systematic summary of ApoA-I expression levels after spinal cord injury, and there are few relevant interventions. An ApoA-I peptide mimetic made from D-amino acids, D-4F, improved HDL-mediated cholesterol efflux from macrophages and reduced foam cell formation in spinal cord injury (113). Notably, given the synergistic role that ABCA1 and ABCG1 proteins play with ApoA-I and, as subsequently described, ApoE during lipid efflux, their simultaneous intervention may have a better effect on foam cell formation and accumulation.

#### 4.2.3 Apolipoprotein E

ApoE is a well-characterised lipoprotein that is expressed in several organs of the body, with the highest levels of expression in the liver, followed by the CNS, and it can also be synthesised by other tissues including monocytes (such as macrophages). The main cell type expressing ApoE in the CNS is astrocytes, while microglia also express it to some extent (114, 115). ApoE is involved in various activities including lipid transport, synaptic growth and neuroplasticity, and has been shown to play an important role in a variety of neurological diseases (116–120). Previous studies have shown that APOE4 is associated with poorer neurological recovery and longer rehabilitation time in patients with traumatic cervical spinal cord injury (121), s suggesting a potential role for ApoE in spinal cord injury. ApoE expression was significantly increased during the subacute phase of traumatic spinal cord injury in mice (122, 123). Single-cell RNA sequencing showed that ApoE was the most up-regulated gene expressed in macrophages and microglia during the subacute and chronic phases, and also ApoE was a hub gene for macrophages and microglia during the subacute and chronic phases of SCI (124). These results suggest that ApoE plays an important role in macrophages and microglia in the subacute and chronic phases of SCI. Deficiency of ApoE exacerbates the inflammatory response and mediates poor functional recovery after spinal cord injury (122, 124, 125), which is related to the leakage of macromolecules, such as inflammatory cells and immune proteins, from the blood-spinal cord barrier due to the increased permeability caused by the ApoE deficiency, and the direct effect of ApoE on the immune response. ApoE is the main cholesterol carrier involved in lipid efflux from CNS cells (126). Under normal conditions, the ABCA1 transporter protein interacts with lipid-poor/free ApoA-I, which acquires cellular PL and FC to form nascent HDL and initiate the RCT (127). imilarly, in the CNS, ABCA1 interacts with lipid-poor/free ApoE to form HDL that is structurally distinct from ApoA-I-HDL (128), and it is recognised that HDL formed by different ApoE isoforms (ϵ2, ϵ3 and ϵ4) have different roles in the CNS, which has been particularly evident in studies related to Alzheimer’s disease (129). Transmission electron microscopy observations revealed an increase in the number of lipid droplets and dense lysosomal material in macrophages and microglia in ApoE-/- mice (124), suggesting that ApoE may be a therapeutic target to promote the restoration of lipid metabolism and to reduce foam cell formation after spinal cord injury. Notably, the molecular size and structure of ApoE dictate that it is difficult to cross the blood-brain barrier, and thus exogenous ApoE mimetic peptides have been developed for use as alternative therapies, which retain the anti-inflammatory and neuroprotective effects of natural ApoE proteins but can still cross the blood-brain barrier (120, 130). The neuroprotective effects of exogenous apoE mimetic peptides have now also been demonstrated in several models of neurological disease including spinal cord injury (116, 120, 124, 131–133). In conclusion, endogenous ApoE has important neuroprotective effects after spinal cord injury and exogenous apoE mimetic peptides may be a new promising neuroprotective agent with the potential to inhibit foam cell formation after spinal cord injury.

## 5 Discussion

After spinal cord injury, the clearance of tissue debris is important for the inflammatory process and the nerve repair process. However, the clearance of tissue debris is hindered by the formation of foam cells. Therefore, the effective regulation of the phagocytosis and metabolic profiles of MDMs for the smooth clearance and metabolism of tissue debris is a key issue for promoting nerve repair after spinal cord injury. We propose two intervention strategies targeting foam cells: (I) reducing the phagocytosis of lipids by macrophages: the phagocytosis of lipid-rich tissue debris (including but not limited to myelin debris) leads to lipid accumulation in macrophages, and this can be improved by regulating or knocking down the relevant lipid uptake receptors (e.g., CD36, CD204, LOX-1); (II) regulating the RCT of macrophages: this process mainly involves ApoA-I, ApoE, and ABCA1 and ABCG1. Accelerating the lipid efflux from macrophages can reduce the excessive accumulation of intracellular lipids and thus prevent the transformation of macrophages into foam cells. It should be noted that the intervention strategies for foam cells are not limited to the ones proposed here; for example, since CE can only be transported to the extracellular compartment after being hydrolysed to FC, the hydrolysis of CE in macrophages limits the cholesterol efflux to some extent, and thus the intervention of related enzymes may also have a positive effect; moreover, since FC available for efflux is mainly transported to the autophagosome via CE in the autophagosome translocation to lysosomes to be supplied by hydrolysis by acid lipase rather than by hydrolysis by neutral cholesterol esterase, the induction of macrophage autophagy or the modulation of lysosomal function may also be effective intervention strategies. Traditionally, foam cell formation has been mainly linked to cholesterol uptake via natural and modified LDL, and the high cholesterol levels in the microenvironment due to the unique lipid composition of myelin sheaths after spinal cord injury have led to the article’s focus on cholesterol efflux. However, other lipids, such as nonesterified fatty acids and triacylglycerol (TAG)-rich lipoproteins (very low-density lipoproteins and chymotrypsin), can also interact with macrophages. Moreover, the intracellular composition of lipid droplets in foam cells after spinal cord injury and the proportion of lipid components phagocytosed and accumulated by macrophages are unclear, as there is a lack of analysis and studies on these aspects. Therefore, besides the foam cell treatment methods summarized in this article based on cholesterol efflux, we encourage researchers to explore more diverse approaches to foam cells after spinal cord injury. In conclusion, although many studies have obtained positive results, further studies are required to understand the differential effects of foam cell intervention and its potential as a pharmacological target for SCI repair.

## Author contributions

XW: Conceptualization, Validation, Visualization, Writing – original draft, Writing – review & editing. ZL: Investigation, Software, Writing – review & editing. HD: Conceptualization, Formal Analysis, Software, Writing – review & editing. WL: Conceptualization, Visualization, Writing – review & editing. CZ: Formal Analysis, Project administration, Writing – review & editing. XX: Supervision, Visualization, Writing – review & editing. HK: Writing – review & editing, Resources. RP: Supervision, Writing – review & editing. D–GY: Supervision, Writing – review & editing. JL: Funding acquisition, Supervision, Writing – review & editing. FG: Supervision, Writing – review & editing.
